# Suicide among War Veterans 

**DOI:** 10.3390/ijerph9072504

**Published:** 2012-07-19

**Authors:** Vsevolod Rozanov, Vladimir Carli

**Affiliations:** 1 Institute of Innovative and Post-Diploma Education, Odessa National Mechnikov University, 2 Dvoryanskaya Street, Odessa 65082, Ukraine; Email: rozanov@te.net.ua; 2 National Centre for Suicide Research and Prevention of Mental Ill-Health (NASP), Karolinska Institute, Stockholm 10523, Sweden

**Keywords:** war and military services veterans, mental health problems, suicide, prevention

## Abstract

Studies aiming to identify if war veterans are at higher risk of suicide have often produced inconsistent results; this could be due to the complexity of comparisons and different methodological approaches. It should be noted that this contingent has many risk factors, such as stressful exposures, wounds, brain trauma and pain syndrome. Most recent observations confirm that veterans are really more likely to die of suicide as compared to the general population; they are also more likely to experience suicidal ideation and suffer from mental health problems. Suicides are more frequent in those who develop PTSD, depression and comorbid states due to war exposure. Combat stress and its’ frequency may be an important factor leading to suicide within the frame of the stress-vulnerability model. According to this model, the effects of stress may interact with social factors, interpersonal relations and psychological variables producing suicidal tendencies. Modern understanding of stress-vulnerability mechanisms based on genetic predispositions, early life development, level of exposure to stress and stress-reactivity together with interpersonal aspects may help to build more effective suicide prevention programs based on universal/selective/indicated prevention principles.

## 1. Introduction

When discussing suicides in specific occupational or social groups, several main questions usually arise: (1) are representatives of the specific group at higher risk of suicide compared to the rest of population?; (2) are there any specific features of environmental or social processes within these groups, which may impact suicide rates?; (3) can targeted prevention programs be implemented within this framework and is it possible to improve its potential efficacy? 

In this review, we are aiming to better understand the potential risk factors and pathways leading to suicides among war veterans. This does not mean all military. Systematic analyses regarding suicide in various national militaries show (*i.e.*, with very rare exceptions) that suicide rates are lower in the military when compared to corresponding age and gender groups from the general population [1,2,3]. Thus, being in the armed forces does not necessarily mean higher risk of suicide. Today, the military setting is quite different from what was observed and extensively discussed (*i.e.*, while describing altruistic suicide) by Durkheim in his classical work. Focus has shifted to specific military persons who have experienced stressful events in certain hostile environments, e.g., modern war zones. Thus, former military that are discharged after being at war (referred to as war veterans) is an important topic, not only concerning suicide, but regarding psychological and emotional consequences of war as well. To put in a broader context, the military is the subject of discussion concerning mental health consequences of war exposures, wherein suicide is one of the most striking [4,5,6]. It must be noted that war veterans constitute only a part of the general heterogeneous veterans’ population. This more general contingent may be at higher risk too, and after persons are discharged from the military, it is important to access the psychosocial implications upon returning to civilian status. On the other hand combat stress and being directly in a war zone, as can be seen further, seems to play the most important role. 

There is no consensus in the expert community regarding suicide rates among veterans of military services. Different studies have produced conflicting results; this is potentially due to the different time periods, methodology and socio-cultural contexts implemented. On the other hand, mental health problems among those who have been involved in war conflicts attract more attention; efforts are taken by governments, public organizations and veterans’ associations to prevent suicides in this contingent. This is especially true for USA where alarming news of growing suicide rates in the Army are being disseminated. Despite prevention measures in the military, the number of suicides continues to increase [7,8,9,10]. Quite recent analyses from USA definitely answered “no” to the question “Is there an epidemic of suicides among current and former US military personnel?” [11,12]. We can confirm this conclusion, especially in regards to inappropriate formulations used by mass media. But, the same authors, who are monitoring the situation in USA for many years, consider that the most recent increased risk of suicide among war veterans warrants attention [11]. Suicides in war veterans are a sensitive topic and emerging knowledge from current and previous research should be reviewed occasionally to provide a more objective basis for prevention measures and this may be important for different countries.

It is suggested (confirmed in many studies) that there are multiple risk factors among military persons when faced with civilian life after retirement and combat exposures. These individuals (mostly males, though this trend appears to be shifting) often enter into middle age carrying the burden of stressful war experiences; being very familiar with firearms, with growing alcohol problem, higher risk for physical health problems due to previous trauma, pain syndrome, often facing family conflicts, social isolation *etc*. [13,14]. On the other hand the majority of military suicides occur among young males, shortly after being discharged from military service. Thus, this raises the question of why suicides are becoming more common among younger veterans. We consider that this, as well as many other specific features of veteran suicides, can be understood within the “stress-vulnerability model” [15].

This model is based on bio-psychosocial synthesis encountering complex relations between genetic predispositions, personality dimensions and deteriorating (or protective) influences of the social environment. From this point of view, environmental stressful exposures throughout life play a very important role. There is an extensive body of evidence showing that childhood abuse and adult adverse exposures may provoke psychopathology such as mood disorders, PTSD or alcohol and drug abuse and dependence, thereby, leading to suicide [16,17,18]. At every stage of developmental trajectory, *i.e.*, from prenatal state and early childhood to middle and older age, stressful life events interact with genetic predispositions and produce adaptive or maladaptive behavioural, emotional and cognitive responses, possibly through epigenetic mechanisms that establish higher reactivity of stress-systems of the organism [19,20,21]. This concept also complies with recently published explanations of growing suicides among war veterans, such as Interpersonal Theory of Suicide, which gives valuable insight in deeper psychological mechanisms [22]. 

## 2. Methodology

An electronic literature search was conducted using the MEDLINE database. Papers published in English were selected. There were no restrictions on publication date or publication status. The identifiers used were “suicides in war veterans”, “suicides in the military” and “military mental health”, combined with the identifiers “suicide*”, or “suicidal behavior” or “self-injurious behavior”. Papers were included in the review if they met the following inclusion criteria: discussed issues as suicidal behaviour or suicide prevention among war veterans, former peacekeepers or representatives of any other militarized occupations. Papers were independently and systematically reviewed by experienced researchers. According to the inclusion criteria, 97 relevant publications were identified on the basis of the title and the abstract. The full-text of 57 papers was available and retrieved for further analysis. Studies were assessed regarding study type, study population, methodology and outcome measures. The heterogeneity of study populations, and the methodology, ruled out formal meta-analysis, therefore a narrative synthesis is presented.

## 3. Results and Discussion

Though suicides in the military have been depicted for centuries, suicide among retired war veterans started to attract more attention in the USA shortly after the Vietnam War [23,24]. Initial studies in this field have not confirmed higher suicide rates in this group; however, it has been noted that males, who have been at war, tend to commit suicide at a younger age compared to the general population. Research indicates that the presence of mental illness leads to a nine-fold increase in suicide rates [24]. After the inclusion of Posttraumatic Stress Disorder (PTSD), as a new diagnosis in the DSM-III, the awareness of this problem substantially increased [25]; this perhaps was the result of intense investigations concerning the mental state of those who have undergone war. Studies among veterans from the Vietnam War have shown that there is a direct causal link between traumatic stress exposure and subsequent PTSD [26]. Moreover, those Vietnam veterans who had a diagnosis of PTSD were shown to have a significantly (~4-fold) increased standardised mortality rate of suicide [27]; those who had been wounded were also at higher suicide risk [28]. In other studies, Vietnam veterans were shown to have increased levels of suicidal ideation and a history of suicide attempts; this was also highly correlated with a PTSD diagnosis. With additional diagnoses, particularly depression, there was an even stronger association [29,30]. However, after the Vietnam studies, research began to focus on the Gulf War veterans. The risk of suicide in this cohort did not increase in comparison to other veterans, who did not take part in the war, nor the general population [31,32]. 

In a new era, conflicts in Iraq and Afghanistan have involved bigger contingents of military from different countries (mostly from USA) and for longer period of time. Suicides in veterans, tracked by different official and public structures and agencies, have become an important public health concern, and it continuously attracts the attention of mass media, politicians and civil society. In the USA, “record high” suicide rates have been recently reported in both the armed forces (*i.e.*, higher than the general population for the first time in history) and in military personnel returning from war [10,33]. An important landmark comprised a publication indicating that veterans returning from Iraq and Afghanistan war zones had significantly higher standardized mortality ratio (SMR) compared to the general population [34]. In another important study, about 104,000 of veterans comprising different ages from Vietnam and post-Vietnam conflicts were compared with more than 200,000 non-veterans Results confirmed that the risk of suicide among war veterans was higher than non-veterans [13]. However, other research (using different access methods) that analysed mortality rates among some 500,000 participants in the Cancer Prevention Study concluded that the risk of death by suicide among middle-aged and older US males was independent of veteran status [14]. Several other studies found that suicide mortality in war veterans’ cohorts are not higher in males compared to the general population; however, all-cause mortality, vehicle-related and drug-related mortality (often suspected as hidden suicides) were higher among the non-veteran group [35,36]. Strong evidence coming from most cited studies [13,34] leaves the impression of a growing problem, especially in USA. It may also reflect the special situation found in USA. Among Australian veterans of the Vietnam War, suicide mortality rates did not significantly differ compared to the general population and non-veterans [37]. Similar findings were reported regarding UK veterans who took part in the Gulf War [38]. However, the majority of publications regarding suicide among war veterans derives from USA; studies performed by American authors often aim to evaluate explicit risk factors and suggest more preventive strategies. 

In Table 1, relevant studies are summarized, which provide the possibility for an evaluation with a historical perspective. 

Although there are conflicting results in this field, the studies included in this systematic review suggest that war veterans are at higher risk of suicide compared to the general population. Recent evidence has also underscored the importance of PTSD as an underlying risk factor of suicide. This raises the question on what may be the reasons behind the recent reports on the association between PTSD diagnoses and suicide rates. Do young military personnel have higher risks and vulnerabilities which are further exacerbated by military service or is it specifically connected with the changing military situation? Is it due to the fact that in recent mission persons are faced with longer stays in the war zone, growing number of service tours (*i.e.*, 6-month periods of war conditions), increasing stressful exposures in war zones? Are we facing growing feelings of guilt, existential crises or other complex problems of transition to civilian life due to changing socio-economic situation?

**Table 1:** Studies on suicidality among war veterans.

Author(s)	Country	Contingent	Main results
Pokorny, A., 1967 [23]	USA	Total veterans from the state of Texas for the year 1960, N = 1,154,458	Suicide rates in veterans do not differ from suicide rates of the same age and sex groups from general population.
Adena, M.A.; Cobbin, D.M.; Fett, M.J.; *et al*., 1985 [37]	Australia	All national servicemen, 19,205 of them served in Vietnam (war veterans) compared to 25,677 who served only in Australia (non-veterans) and to Australian population.	Number of deaths of veterans and of non-veterans was less than expected from Australian population, when veterans were compared with non-veterans there was no statistically significant difference in deaths.
The CDC Vietnam Experience study, 1987 [24]	USA	Cohort of 9,324 Vietnam veterans compared to 8,989 veterans who served in Korea, Germany or USA randomly selected from ~5 million USA veterans from national database.	Total mortality in the 5 years after discharge was 17% higher in Vietnam veterans, excess mortality was due to external causes (including suicide).
Breslin, P.; Kang, H.K.; Lee, Y.; *et al*., 1988 [35]	USA	Mortality among 24,235 US Army and Marine Corps Vietnam veterans were compared with that of 26,685 non-Vietnam veterans.	Excess deaths were observed among Army Vietnam veterans for motor vehicle accidents, non-motor vehicle accidents, and accidental poisonings. Suicides were not elevated.
Bullman, T.A.; Kang, H.K., 1994 [27]	USA	4,247 Vietnam veterans from Agent Orange Registry with PTSD diagnosis compared with 12,010 veterans without PTSD.	Veterans diagnosed with PTSD were 3.97 times more likely to die of suicide (SMR was 6.74 for veterans with and 1.67 for those without PTSD).
Bullman, T.A.; Kang, H.K., 1996 [28]	USA	Risk of suicide for 34,534 veterans who were wounded in Vietnam was evaluated with regards of severity of wound and number of times being wounded.	In comparison with the US male general population, veterans hospitalized due to combat wound or wounded more than once had a significantly increased risk of suicide (SMR 1.22 and 1.58 respectively).
Kang, H.K.; Bullman, T.A., 1996 [31]	USA	Retrospective cohort study of postwar mortality among 695,516 Gulf War veterans and 746,291 other veterans followed through September 1993.	There was a small but significant excess of deaths in the Gulf War veterans mainly caused by accidents. No difference in suicides was found (RR = 0.94).
Macfarlane, G.J.; Thomas, E.; Cherry, N., 2000 [38]	UK	A retrospective cohort study including all 53,462 UK Gulf War veterans in comparison to equivalent cohort of personnel who were not deployed.	Mortality from “external” causes was higher in the Gulf cohort (mortality rate ratio 1.18) however, no excess of deaths were recorded as suicide.
Boehmer, C.; *et al*., 2004 [36]	USA	Mortality of 9,324 Army veterans was compared to 8,989 non-Vietnam veterans from the date of discharge the end of the year 2000.	The crude RRs for suicides and homicides were somewhat elevated in the first 5 years (1.72) but not in the next 5 years of follow-up (0.93).
Boscarino, J.A., 2006 [43]	USA	7,924 “Vietnam theater” veterans were compared to 7,364 “Vietnam era” veterans with and without PTSD ~30 years after their military service.	Veterans diagnosed with PTSD had an increased risk of death from multiple causes, especially from external, which included suicide, hazard ratio 2.3.
Zivin, K.; Kim, M.; McCarthy, J.F.; *et al.*, 2007 [44]	USA	A retrospective cohort study of 807,694 veterans from the National Registry of Depression for the period of 1999–2004.	0.21% of veterans completed suicide during the follow-up, younger and older veterans were at higher risk than middle-aged ones, those with PTSD had overall lower risk of suicide.
Kaplan, M.; Huguet, N.; McFarland, B.H.; *et al*., 2007 [13]	USA	Prospective follow-up study, sample of 320,890 persons from National Health Interview Survey, among them 104,026 veterans.	Over time veterans were twice as likely (adjusted hazard ratio 2.13) to die of suicide compared with male nonveterans in the general population.
Kang, H.K.; Bullman, T.A., 2008 [34]	USA	Veterans were identified by the Defense Manpower Data Center. The final cohort included all 490,346 veterans who served in Iraq (OIF/OEF) and were separated alive from active duty between October 2001 and December 2005.	The overall risk for suicide was not significantly elevated (SMR 1.15) but was increased for former active duty veterans (SMR1.33) and for veterans diagnosed with a selected mental disorder (SMR 1.77). The most common methods of suicide were by firearm (73%) and by hanging (21%).
Gutierrez, P.M.; Brenner, L.A.; Huggins, J.A., 2008 [57]	USA	114 acute psychiatric admissions, 22 veterans with a history of traumatic brain injury, measured suicidal ideation, nature of suicide attempts.	From post-traumatic patients 6 (27.3%) has made a total of 14 suicide attempts.
Jakupcak, M.; Cook, J.; Imel, Z.; *et al*., 2009 [42]	USA	Iraq and Afghanistan War veterans (N = 407) referred to Veterans Affairs mental health care.	Veterans with PTSD were more than 4 times as likely to endorse suicidal ideation. Risk for suicidal ideation was 5.7 times greater in veterans who screened positive for two or more comorbid disorders.
Miller, M.; Barber, C.; Azrael, D.; *et al.*, 2009 [14]	USA	Prospective cohort study of 499,356 male participants in the Cancer Prevention initiative.	In age-adjusted analyses the risk of suicide did not differ by veteran status.
Pfeiffer, P.N.; Ganoczy, D.; Ilgen, M.; *et al*., 2009 [56]	USA	887,859 patients with depression from Veterans Administration databases.	Odds of completed suicide were significantly increased for patients with panic disorder (OR 1.26) and who received any antianxiety medication (OR 1.71). Odds of completed suicide were decreased among patients with comorbid posttraumatic stress disorder (OR 0.87).
Kaplan, M.S.; McFarland, B.H.; Huguet, N., 2009 [55]	USA	Data from 28,534 suicide decedents from the 2003 to 2006 National Violent Death Reporting System.	The male and female veteran suicide decedents were, respectively, 1.3 and 1.6 times more likely to use firearms relative to nonveterans.
Ilgen, M.A.; Zivin, K.; Austin, K.L.; *et al*., 2010 [59]	USA	Analysing medical records and National Death Index (N = 260,254).	Veterans with severe pain were more likely (hazards ratio 1.33) to die by suicide than patients experiencing none, mild or moderate pain.
Brenner, L.A.; Ignacio, R.V.; Blow, F.C., 2011 [58]	USA	Individuals who received care at US Veterans Administration between fiscal years 2001 to 2006, all patients with a history of traumatic brain injury (n = 49,626).	Veterans with a history of TBI were 1.55 times more likely to die by suicide, those with concussion/cranial fracture were 1.98 times, and those with contusion/ /traumatic intracranial hemorrhage were 1.34 times more likely to die by suicide.

It must be taken into consideration that long-term psychological consequences of war and traumatic stress, in general, can be seen across war veterans’ globally. Mental health among war veterans is very similar across cultures, notwithstanding socioeconomic differences. Suicide rates are usually not calculated and compared to the general population; however, some research has been performed on Russian veterans (*i.e.*, Afghanistan and Chechnya), British Falkland conflict veterans, and Croatian local war veterans [39,40]. Deahl and co-authors [6] suggest that mental health problems of ex-military are diverse, polymorphic and have psychosocial consequences like social exclusion, homelessness, self-harm and substance abuse. In the longitudinal study of male veterans with a history of drug abuse, it was found that PTSD, drug-dependence, non-fatal attempted suicides and suicidal ideation had a strong continuity over time [41], with suicidal ideation some 4-times more frequent in PTSD-sufferers [42]. Veterans diagnosed with PTSD have an increased risk of death, not only from suicide, but from other causes as well [43]. Evidence shows that suicide rates are higher among older and younger depressed veterans with PTSD compared to middle-aged veterans [44]. Moreover, younger males were shown to have the highest suicide risk [23]. 

Among former military personnel, there are many publications that focus on the role of underlying disorders in the genesis of suicidality, especially PTSD. PTSD is a highly comorbid disorder with a growing prevalence among war veterans. PTSD is commonly present in those veterans with a previous history of suicide attempts, psychopathology and severe substance abuse characteristics [45,46]. Aggressive and self-aggressive behaviour in veterans occurs more often in PTSD cases [47]; veterans diagnosed with PTSD have 4-times as many firearms as non-veterans [48]. These data provide an explanation for many veteran suicides; however, suicide cannot be explicated as the result of one particular mental disorder. Moreover, as can be seen from Table 1 some studies do not confirm that those war veterans who suffer PTSD are more likely to commit suicide. Notwithstanding, depression is considered the leading underlying disorder for suicide and recent studies suggest that PTSD has a pronounced effect on risk mostly if comorbid with depression. This issue was addressed by L. Sher, who indicated a significant comorbidity between PTSD and major depressive disorder (MDD) [49]. Recently, the same author proposed that the majority of individuals diagnosed with comorbid PTSD and MDD have a psychobiological condition that can be termed as “posttraumatic mood disorder” (PTMD). The author described studies suggesting that patients suffering from comorbid PTSD and MDD differ clinically and biologically from individuals with PTSD alone or MDD alone. Individuals with comorbid PTSD and MDD are characterized by a greater severity of symptoms, increased suicidality, and a higher level of impairment in social and occupational functioning [50]. Taking into account the “many faces” of male depression (aggression, alcohol abuse, poor social functioning) leading to male vulnerability and gender related public health paradoxes [51], this idea looks very attractive. Logically the model of suicidal behavior in war veterans with PTMD has been proposed by L. Sher which consists of the following components: (1) genetic factors; (2) prenatal development; (3) biological and psychosocial influences from birth to mobilization/deployment; (4) mobilization/pre-deployment stress; (5) combat stress, traumatic brain injury, and physical injury; (6) post-deployment stress; (7) biological and psychosocial influences after the deployment; (8) trigger (precipitant) of a suicidal act; and (9) suicidal act. The first four components, according to the proposed model, determine the vulnerability to combat stress; whereas, the first seven components ascertain a predisposition to suicidal behavior, which is a key element that differentiates PTMD patients with high- and low-risks of suicide [49,50]. Concerning the three key components of the suicidal process (*i.e.*, biological, personality/psychological and social/environmental), the model proposed by Sher is congruent with the more general stress-vulnerability model [9] and takes into account specific social interactions and stressors of the military.

Modern challenges of growing war conflicts, and its potential for comorbid psychopathologies [4,5,6,52], should be taken into consideration as a warning for exacerbating suicides, suicide attempts and psychopathology among newly released military personnel. This has been underscored in recent studies. Janet Wiener and colleagues have followed more than 10,000 veterans, who were hospitalized at the Veterans Affairs medical centre between 1993 and 1998 following a suicide attempt. Results showed that suicide accounted for 13% of all deaths in the study cohort, while in the general population, it accounted for only 1.8% of all deaths [53]. There was a high prevalence of diagnosed alcohol disorder or abuse (31.8%), drug dependence or abuse (21.8%), psychoses (21.2%), depression (18.5%) and hypertension (14.2%) in this cohort. Moreover, veterans who attempted suicide had a substantially higher mortality rate compared with the non-suicide, e.g., heart diseases, cancer and traffic accidents. Thus, mental health problems appeared to be significantly correlated with general health and mortality. The above mentioned study also reported on women suicides among veterans. Among female suicide attempters, completed suicide appeared to be the leading cause of death, while in males, it was second leading cause. This is confirmed in another study, which examined some 6,000 female suicides; results revealed that women aged from 18 to 35 years, who served in the US military, committed suicide nearly three times more frequent than nonveteran females of the same age group [54]. Veterans were more likely to use firearms for suicide compared to nonveterans, even after adjusting for age, marital status, race and region of residence; this applied to both males and females [55], which changes our general view on the violent death as a typical male phenomenon.

Thus, not PTSD alone as an important underlying disorder but also its comorbidity with other psychopathologies and physical health problems serve as factors leading for suicide. Besides the proposed MDD condition, there is evidence indicating that the odds of completed suicide are significantly increased in depressed patients with generalized anxiety disorder and panic disorder [56]. Veterans may also have additional risk factors for suicide, mainly due to brain trauma and pain syndrome. Several publications testify that cognitive, psychiatric and emotional facets of traumatic brain injury are risk factors of attempted and completed suicide [57,58]. Severe pain also predicts higher suicide risks, and in those veterans who are living in institutions, pain and physical illness were the most severe triggers [59,60]. It must be also noted that general health problems add to previous stressful exposures and constitute additional risk factors.

That the level of stressful exposures determines, in many ways, future mental problems and suicidality among veterans can be seen from data regarding peacekeepers. Recently, with the wide role of peacekeeping duties worldwide, several researchers have studied suicide in former peacekeepers [61,62,63,64]. The peacekeeping soldier is not expected to be engaged in regular war activities, rather acts as a buffer between hostile parties. This task is completely different from soldiers who are traditionally trained for combat [3]. This specific group may suffer from serious tension and psycho-emotional problems in regions with different ethnicity and conflicts, but they are usually less exposed to combat stress. Although an increase in suicide by firearms was reported in Norwegian peacekeepers [61,62], a Canadian study was unable to confirm a higher risk of suicides in this group [63]. In a Swedish study performed on 39,768 former peacekeepers, even a lower number of suicides were found compared to the general population [64]. Thus, there is no definite conclusion with more evidence of now rise of suicides. It is possible to speculate that absence of direct combat exposure makes this group different. 

Discussion regarding mechanisms of suicide among war veterans is not limited to psychobiological aspects. It should be noted that a valuable insight into more subtle psychological mechanisms of the problem was presented within the Interpersonal-Psychological Theory of Suicide [22], which is based on an earlier Suicidal Mode discourse [65]. This concept is putting forward three important factors, which may lead to suicide: (1) feelings that one does not belong with other people (thwarted belongingness); (2) feelings that one is a burden on others or society (perceived burdensomeness); and (3) an acquired capability to overcome the fear and pain associated with suicide (acquired capability of lethal self-injury) [22]. Within the frame of this theory many psycho-social aspects concerning discharge from the armed forces (*i.e.*, loosing relations with comrades, problems during the transition period, and feeling excluded from civilian life) are relevant. Stressful experiences of being in the war zone, together with elements of extensive training are also important underpinnings of suicide risks. During military missions, solders may develop certain aggressive and impulsive behavioural automatisms in order to deal with the stressful environments. Combat training and military exposures may cause habituation to fear of painful experiences, including suicide [22]. This evidence adds to the better understanding of veterans’ suicide. Veterans who commit suicide are a heterogeneous group, wherein they are differentially exposed to social, psychological or psychopathological risks. Combat stress and direct experiences of war may be the unifying factor though this factor is valid only for the part of the released military. On the other hand this particular exposure may be the main contributing risk factor that influences suicide rates in a more general contingent. This again emphasises that stress is potentially the main mechanism exacerbating existing vulnerabilities and may serve as the main component for inducing suicide. 

Discussion on suicide prevention measures among veterans, its implementation and effectiveness may be a theme for a specific review. Quite recently, such systematic reviews have covered practically all aspects of this subject, which makes our task much easier [66]. Suicide prevention measures in war veterans vary from systemic policy on the governmental level [8,67] to more local initiatives [68]. Recently, several publications have discussed priorities and main strategies within this field [67,69]. In many cases, the so-called “gatekeepers’ training” strategy is promoted [69,70]. This same strategy was utilized in the management of suicidal crisis in a large military unit in the Ukraine, during the period of severe economic problems and downsizing [71]; results revealed promising outcomes. In USA, the system of prevention is mostly developed within veterans’ health facilities; the main strategies are based on screening for mental health problems, identifying appropriate treatment, and hotlines and awareness training for staff and families [67,68,69,70,71]. The above mentioned systematic review on suicide prevention programs both for military and veterans reports that there are insufficient data, thus, concrete conclusions regarding the effectiveness of these programs are ambiguous [66]. Of course, this should not discourage those who are involved in these activities, as the effectiveness of these programs cannot be definitively measured. Thus, each saved life is a valuable achievement.

## 4. Conclusions

This review attempts to understand why suicide is becoming more noticeable among war veterans. Returning back to the questions formulated in the beginning of this paper, we can conclude that war veterans definitely have specific social and environmental factors that may increase their risks for suicidality. On the other hand, it is difficult to draw a definitive conclusion regarding higher suicide rates among veterans when compared with the general population. This group is very heterogeneous. Veterans of different age groups and involved in different conflicts may vary in risk, thus, appropriate comparisons is essential. It is not always feasible so far as studies on veterans’ suicides are using different access methods, cover different time periods and the level of war exposure cannot be easily identified within studied cohorts. On the other hand those who were involved in latter conflicts, as in Iraq, are definitely at higher risk [11,34,72]. Even with the “healthy soldier effect”, good mental and physical health is not always protective from adverse effects of war exposures and subsequent deaths from suicides [73]. This may be well understood within the stress-vulnerability model, which integrates biological and psychosocial factors. It also explicates the interaction of different kinds of stress as a pathway to suicidal behavior. On the other hand, many aspects of this phenomenon may lie in deep psychological and existential problems of those who were involved in the wars that USA and other countries are leading abroad. The problem of the wish to die cannot be understood only in terms of traditional risk factors. Those returning from war may also experience changes in identity and alienation from civilian life [74]. Complex interpersonal problems and acquired capability to overcome fear of extreme pain may contribute to suicidal tendencies in war veterans as well [22,75]. 

In spite of discrepancies between different studies, the general conclusion is very likely to confirm that war veterans have many mental health problems [5,6,52,72,76] and suicide is the clearest one among them. Many studies concentrate on PTSD as an underlying disorder, nevertheless some authors point that PTSD has a pronounced effect on suicide risk especially in comorbidity with depression. On the other hand psychiatric disorders (though being important predictors of suicide) are not able to explain all suicides in veterans. It should be taken into consideration suicide is often a pathway for alleviating pain and suffering which may be independent from diagnoses. It is not even so important if suicide rates in veterans are slightly higher or lower than in the general population. It is more important to consider the moral responsibility of society towards those who are facing numerous psychological and health problems after serving the military [77]. It is also a very vivid example of how stressful environmental exposures can reveal hidden vulnerabilities, thus, producing mental health problems. Moreover, aggressive and impulsive components of the training usually applied to modern combatants may lead to negative consequences in civilian life after discharge. There is no doubt that many interdisciplinary studies are needed to better understand stress vulnerability and resilience in war veterans, including genetic/epigenetic, psychological, cognitive, social and spiritual factors. 

For development of preventive measures, we rely on known approaches in suicide prevention. The universal/selective/indicated model [78] seems to be the most relevant. In general terms, the universal prevention is aimed at the general population (*i.e.*, those enrolled in the armed forces in our case), selective is aimed at those who have an elevated risk (e.g., combat veterans) and indicated, which is aimed at addressing the symptoms (*i.e.*, those who have developed PTSD or mood disorders, experienced brain trauma, have been wounded causing residual pain syndrome, or appeared in a situation of crisis, alienation and/or exclusion). From this point of view, suicide prevention in war veterans should focus on (1) better screening at admission to the Army (e.g., early life events should be addressed more seriously, and possibly in future genetic variables should also be taken into consideration); (2) development of better strategies of psychological training (e.g., stress-inoculation) and support; (3) implementation of veterans’ status strategies that are based on improvement in recognition of mental health problems and better treatment of comorbid psychiatric conditions; (4) better control of physical health, rehabilitation from trauma and pain management; (5) better identification of suicidal tendencies; and (6) social and family support, social inclusion, measures to overcome thwarted belongingness and to decrease one's sense of burdensomeness, as well as possible counterbalancing acquired capability. Many parts of these strategies are already being implemented, however, have not been thoroughly evaluated. A combination of state-of-the-art research concerning developmental trajectory and stress-vulnerability acquirement via genetic and epigenetic mechanisms are also needed. 
